# Prevalence of Non-Alcoholic Fatty Liver Disease by Ultrasonographic Assessment in Lean Individuals With Diabetes

**DOI:** 10.7759/cureus.116718

**Published:** 2026-09-22

**Authors:** Nikhil K D. G., Manana Gowda, Madhusudan Pai

**Affiliations:** 1 Department of General Medicine, S.S. Institute of Medical Sciences and Research Centre, Davangere, IND

**Keywords:** glycated hemoglobin, hepatic steatosis, hypertriglyceridemia, lean nafld, type 2 diabetes mellitus, ultrasonography

## Abstract

Background

Non-alcoholic fatty liver disease (NAFLD) is increasingly recognized in individuals with a normal body mass index (BMI), a phenotype termed “lean NAFLD.” Data on its prevalence and association with glycemic control among Indian diabetic populations remain limited. This study aimed to determine the prevalence of NAFLD in lean individuals with diabetes (BMI <23 kg/m²) and to evaluate its association with glycated hemoglobin (HbA1c) levels.

Methodology

This hospital-based observational study was conducted in the Department of General Medicine at a tertiary care center between September 2022 and August 2024. One hundred lean diabetes patients (BMI <23 kg/m²), 86 inpatients and 14 outpatients, diagnosed per American Diabetes Association (ADA) criteria, were enrolled; as all participants had type 2 diabetes mellitus, they met at least one cardiometabolic criterion for metabolic dysfunction-associated steatotic liver disease (MASLD), and the terms NAFLD and MASLD are used interchangeably in this report. Clinical parameters, fasting lipid profiles, and HbA1c were recorded, and abdominal ultrasonography was performed prospectively by a single radiologist blinded to clinical history, diabetes treatment, and laboratory results, to diagnose NAFLD and grade its severity (grades 1-3). Associations were assessed using the chi-square test and independent-samples t-test; all analyses were unadjusted bivariate comparisons.

Results

Of the 100 participants, 48 were male and 52 were female. NAFLD was detected in 38% (n=38; 95% CI 29.1-47.8%) of participants, with a higher prevalence in male patients (n = 21/48, 43.8%; 95% CI 30.7-57.7%) than female patients (n = 17/52, 32.7%; 95% CI 21.5-46.2%). Prevalence was similar between inpatients (32/86, 37.2%) and outpatients (6/14, 42.9%; p=0.69). Among affected participants, grade 1 disease was most common (n = 20/38, 52.6%; 95% CI 37.3-67.5%), followed by grade 2 (n = 14/38, 36.8%; 95% CI 23.4-52.7%) and grade 3 (n = 4/38, 10.5%; 95% CI 4.2-24.1%). Mean HbA1c did not differ significantly between participants with and without fatty liver (8.75±2.26% vs. 8.94±2.18%; mean difference −0.19%, 95% CI −1.10 to 0.71; p=0.674). Fasting blood glucose >130 mg/dL was significantly more common among participants with fatty liver (OR 5.25, 95% CI 1.12-24.57; p=0.022); the association with elevated serum triglycerides (>149 mg/dL) did not reach statistical significance once the test statistic was corrected (OR 1.50, 95% CI 0.67-3.38; χ²=0.96, p=0.33).

Conclusion

NAFLD is common among lean Indian diabetes patients and occurred across a broad range of glycemic control. Fasting blood glucose, but not triglycerides or HbA1c, was significantly associated with NAFLD in this unadjusted analysis. These findings highlight the importance of considering NAFLD in lean individuals with diabetes and support further evaluation of targeted ultrasonographic screening in this population.

## Introduction

Non-alcoholic fatty liver disease (NAFLD) has emerged as a major global health concern, affecting approximately 25% of the adult population worldwide [1]. Its prevalence parallels rising rates of obesity and type 2 diabetes mellitus (T2DM), making NAFLD the most common chronic liver disease. The disease spectrum ranges from simple steatosis to non-alcoholic steatohepatitis (NASH), which may progress to fibrosis, cirrhosis, and hepatocellular carcinoma [2,3].

The pathophysiology of NAFLD is multifactorial, involving genetic, metabolic, and environmental factors. Insulin resistance plays a central role by promoting hepatic lipid accumulation and oxidative stress-induced injury [4]. Genetic variants such as *PNPLA3* and *TM6SF2* also contribute significantly [5-7], while lifestyle factors further influence disease severity [8].

NAFLD carries considerable systemic consequences, increasing the risk of cardiovascular disease, metabolic syndrome, and T2DM [9,10]. In India, reported NAFLD prevalence ranges from 9% to 53%, with higher rates in urban populations [11]. Although commonly linked to obesity, NAFLD also occurs in individuals with a normal BMI, a phenotype termed lean NAFLD. These patients exhibit distinct metabolic and genetic profiles and remain at risk of progressive liver disease despite a normal BMI [12,13]. However, data on lean NAFLD, particularly among Indian patients with diabetes, remain limited, and understanding its prevalence and clinical correlates in this subgroup is important for improving early detection.

Thus, this study was undertaken to: (i) determine the prevalence of NAFLD in lean individuals with diabetes (BMI <23 kg/m²), and (ii) evaluate the association between NAFLD presence and glycated hemoglobin (HbA1c) levels.

## Materials and methods

Study setting and population

This was a hospital-based observational study conducted in the Department of General Medicine at S.S. Institute of Medical Sciences and Research Centre, Davangere, Karnataka, India, between September 2022 and August 2024, and is reported in accordance with the STROBE (Strengthening the Reporting of Observational Studies in Epidemiology) guidelines for cross-sectional studies.

Eligibility criteria

Inclusion criteria were: Individuals aged over 18 years with a BMI <23 kg/m² and diabetes mellitus diagnosed per American Diabetes Association (ADA) criteria (HbA1c ≥6.5%; fasting plasma glucose ≥126 mg/dL; two-hour plasma glucose ≥200 mg/dL; or random plasma glucose ≥200 mg/dL with classic symptoms) [14]. The BMI threshold of <23 kg/m² follows the WHO Asia-Pacific and Asian Pacific Association for the Study of the Liver (APASL) MASLD guideline definition of a lean/normal-weight phenotype in Asian populations, which use lower BMI cut-points than international thresholds because Asian populations develop metabolic complications at lower BMI [15]; no participant in this cohort was underweight (BMI <18.5 kg/m²; observed range 19.35-22.95 kg/m²).

A structured social history was obtained from all participants to assess alcohol consumption; individuals reporting alcohol intake above two units/day for men (≈16 g/day) or one unit/day for women (≈8 g/day) were excluded, along with those with BMI ≥23 kg/m², a history of hepatotoxic drug use, or positive hepatitis B or C serology. Only patients with type 2 diabetes mellitus (T2DM) were included; diabetes type was established on clinical grounds alone (age at onset, absence of a ketosis-prone presentation, and physician judgment) in addition to meeting the ADA glycemic criteria above. Autoantibody (e.g., glutamic acid decarboxylase (GAD)) or C-peptide testing to formally exclude type 1 diabetes or latent autoimmune diabetes of adulthood was not performed, chiefly for cost reasons.

Sample size

Sample size was calculated using the formula $n = \frac{Z^2 \times p \times q}{d^2}$, where Z=1.96 (α=0.05), p=10.3% (expected prevalence of lean NAFLD) [16], q=89.7%, and d=6% (absolute precision), yielding a required sample of 99, rounded up to 100 participants. 

Consecutive lean diabetes patients attending the outpatient department or admitted to medical wards were screened for eligibility against the inclusion and exclusion criteria. Screening and enrollment continued consecutively until the pre-calculated target sample size of 100 was reached. Of 182 patients screened, 76 did not meet the eligibility criteria, and six declined consent, leaving exactly 100 eligible, consenting participants, at which point enrollment was closed. No participant was excluded after enrollment, and no adjustments were made to the composition of the enrolled group to fit the calculated sample size.

Data collection and diagnostic procedures

Written informed consent was obtained from all participants, and clinical history and physical measurements were recorded on a standardized proforma. Fasting blood sugar, postprandial blood sugar, and random blood sugar were analyzed by the hexokinase method (Roche Holding AG, Basel, Switzerland), and HbA1c was measured by high-performance liquid chromatography (Bio-Rad Laboratories, Inc., Hercules, California, United States). Fasting lipid profiles were estimated after an 8-14-hour fast. Duration of diabetes, waist circumference, and specific antidiabetic or lipid-lowering medication classes (glucagon-like peptide-1 (GLP-1) receptor agonists, sodium-glucose cotransporter 2 (SGLT2) inhibitors, insulin, statins) were not systematically recorded on the study proforma and could therefore not be reported.

Participants fasted for at least six hours prior to ultrasonography. Abdominal ultrasonography was performed using a GE LOGIQ P9 system with a 3.5-5 MHz curvilinear transducer (GE Healthcare Technologies, Inc., Chicago, Illinois, United States), with each examination lasting approximately 20 minutes. Fatty liver was graded according to the criteria of Saverymuttu et al. [17] as follows: (i) grade 1 (mild): a slight, diffuse increase in fine hepatic echogenicity with normal visualization of the diaphragm and intrahepatic vessel borders; (ii) grade 2 (moderate): a moderate increase in fine echogenicity with slightly impaired visualization of the intrahepatic vessels and diaphragm; and (iii) grade 3 (severe): a marked increase in echogenicity with poor or absent visualization of the intrahepatic vessel borders, diaphragm, and posterior liver parenchyma. All ultrasound examinations were performed and interpreted prospectively, in real time, by a single radiologist with over 20 years of experience in abdominal sonography, who was blinded to participants’ clinical history, diabetes treatment, and laboratory results. Formal assessment of intra- or inter-observer reliability was not performed, and individual severity grades could not be independently re-verified against the original imaging records; accordingly, the association between NAFLD severity grade and HbA1c is not reported (see Limitations).

Statistical analysis

Data were analyzed using IBM SPSS Statistics for Windows, version 26.0 (IBM Corp., Armonk, New York, United States). Categorical variables were expressed as frequencies and percentages, and continuous variables as mean ± standard deviation (SD). Associations between categorical variables were tested using the chi-square test, with odds ratios (ORs) and 95% confidence intervals (CIs) calculated for each comparison, and continuous variables between two groups were compared using the independent-samples t-test after confirming the equality-of-variance assumption (Levene’s test). A p-value <0.05 was considered statistically significant. All reported associations reflect unadjusted, bivariate comparisons; multivariable analysis to control for potential confounders (age, sex, BMI, diabetes duration, medications, hypertension, and lipid levels) was not performed, and associations should not be interpreted as independent or causal.

## Results

A total of 100 lean diabetes patients (BMI <23 kg/m²) were evaluated. Most participants were aged 51-70 years (56%), with an overall mean age of 60.6±12.9 years; gender distribution was roughly equal (52% female, 48% male), and most participants (58%) had a BMI between 22 and 23 kg/m² (Table 1). No participant had a BMI below 18.5 kg/m² (observed range 19.35-22.95 kg/m²).

**Table 1:** Baseline demographics, comorbidities, and BMI distribution (N=100) BMI: body mass index; T2DM: type 2 diabetes mellitus

Parameter	Subcategory	Frequency (Percentage)	Mean ± SD
Age (years)	≤30	3 (3%)	60.6 ± 12.9
	31–50	22 (22%)	
	51–70	56 (56%)	
	>70	19 (19%)	
Gender	Female	52 (52%)	—
	Male	48 (48%)	
Comorbidities	Hypothyroidism	4 (4%)	—
	Hypertension	51 (51%)	
	Smoking	13 (13%)	
	Newly diagnosed T2DM	19 (19%)	
BMI (kg/m²)	19–20	10 (10%)	21.8 ± 1.05
	20–21	6 (6%)	
	21–22	26 (26%)	
	22–23	58 (58%)	

NAFLD was detected in 38 participants (38%; 95% CI 29.1-47.8%), with a higher prevalence in male patients (43.8%; 95% CI 30.7-57.7%) than female patients (32.7%; 95% CI 21.5-46.2%). Prevalence was similar between inpatients (32/86, 37.2%; 95% CI 27.7-47.8%) and outpatients (6/14, 42.9%; 95% CI 21.4-67.4%; χ²=0.16, p=0.69), supporting generalization of the overall estimate across both settings within this center. The highest proportion of fatty liver was observed among participants aged ≤30 years (66.7%), although this subgroup was small (n=3) (Table 2).

**Table 2:** Distribution of participants with and without NAFLD according to age, sex, and setting NAFLD: non-alcoholic fatty liver disease

Parameter	Subcategory	With NAFLD (n = 38), n (%)	Without NAFLD (n== 62), n (%)
Age (years)	≤30	2 (66.7%; 95% CI 20.8–93.9%)	1 (33.3%; 95% CI 6.1–79.2%)
	31–50	8 (36.4%; 95% CI 19.7–57.0%)	14 (63.6%; 95% CI 43.0–80.3%)
	51–70	21 (37.5%; 95% CI 26.0–50.6%)	35 (62.5%; 95% CI 49.4–74.0%)
	>70	7 (36.8%; 95% CI 19.1–59.0%)	12 (63.2%; 95% CI 41.0–80.9%)
Gender	Female	17 (32.7%; 95% CI 21.5–46.2%)	35 (67.3%; 95% CI 53.8–78.5%)
	Male	21 (43.8%; 95% CI 30.7–57.7%)	27 (56.3%; 95% CI 42.3–69.3%)
Setting	Inpatient	32 (37.2%; 95% CI 27.7–47.8%)	54 (62.8%; 95% CI 52.2–72.3%)
	Outpatient	6 (42.9%; 95% CI 21.4–67.4%)	8 (57.1%; 95% CI 32.6–78.6%)
Overall	—	38 (38.0%; 95% CI 29.1–47.8%)	62 (62.0%; 95% CI 52.2–70.9%)

Participants with fatty liver had significantly higher rates of elevated fasting blood glucose than those without it (Table 3); postprandial blood glucose, HbA1c category, and triglycerides did not differ significantly between groups after correcting the triglyceride test statistic (Table 3). 

**Table 3:** Metabolic parameters according to NAFLD status Categorical variables were compared using the Chi-square test. NAFLD: non-alcoholic fatty liver disease; FBS: fasting blood sugar; PPBS: postprandial blood sugar; HbA1c: glycated hemoglobin

Parameter	Category	With NAFLD (n = 38), n (%)	Without NAFLD (n = 62), n (%)	Test Statistic (χ²)	p-value
FBS (mg/dL)	≤130 (n=16)	2 (12.5%)	14 (87.5%)	5.26	0.022 (OR 5.25, 95% CI 1.12–24.57)
	>130 (n=84)	36 (42.9%)	48 (57.1%)		
PPBS (mg/dL)	≤180 (n=21)	6 (28.6%)	15 (71.4%)	1.003	0.317 (OR 1.70, 95% CI 0.60–4.85)
	>180 (n=79)	32 (40.5%)	47 (59.5%)		
HbA1c (%)	≤7 (n=26)	10 (38.5%)	16 (61.5%)	0.003	0.955 (OR 0.97, 95% CI 0.39–2.44)
	>7 (n=74)	28 (37.8%)	46 (62.2%)		
Triglycerides (mg/dL)	≤149 (n=51)	17 (33.3%)	34 (66.7%)	0.96	0.33 (OR 1.50, 95% CI 0.67–3.38)
	>149 (n=49)	21 (42.9%)	28 (57.1%)		

Among the 38 participants with NAFLD, grade 1 (mild) disease was most frequent (52.6%; 95% CI 37.3-67.5%), followed by grade 2 (moderate, 36.8%; 95% CI 23.4-52.7%) and grade 3 (severe, 10.5%; 95% CI 4.2-24.1%) (Table 4).

**Table 4:** NAFLD severity grade distribution (N = 38) NAFLD: non-alcoholic fatty liver disease

NAFLD grade	Frequency	Percentage (95%CI)
Grade 1 (mild)	20	52.63 (95% CI 37.3–67.5)
Grade 2 (moderate)	14	36.84 (95% CI 23.4–52.7)
Grade 3 (severe)	4	10.52 (95% CI 4.2–24.1)

Continuous variables between the two groups were compared using the independent-samples t-test after confirming equal variances (Levene’s test: F=0.03, p=0.86). The presence of fatty liver did not differ significantly by HbA1c category (≤7% vs. >7%, p=0.955) (Table 3) or by overall mean HbA1c (8.75±2.26% vs. 8.94±2.18%, p=0.674) (Table 5).

**Table 5:** Comparison of mean HbA1c between participants with and without NAFLD Continuous variables were compared using the independent-samples t-test. HbA1c, glycated hemoglobin; NAFLD, nonalcoholic fatty liver disease; *SD*, standard deviation; *t*, t statistic; *df*, degrees of freedom

Group	Frequency	Mean HbA1c (%)	SD	t	df	p
With NAFLD	38	8.75	2.26	-0.422	98	0.674
Without NAFLD	62	8.94	2.18			

## Discussion

This study evaluated the prevalence of NAFLD among lean individuals with diabetes (BMI <23 kg/m²) and examined its correlation with glycemic control. An overall NAFLD prevalence of 38% in lean diabetic patients was found, indicating that hepatic steatosis is a frequent comorbidity even in non-obese diabetic cohorts. This exceeded the 10.3% prevalence assumed in the a priori sample-size calculation, which may reflect differences in population characteristics or diagnostic thresholds between studies. This finding is consistent with the wider Asian literature suggesting that lean NAFLD involves metabolic derangements that extend beyond body mass index alone [1,12,13]. It is further supported by contemporary data on BMI thresholds and adverse hepatic outcomes in lean steatotic liver disease across East Asian populations, which similarly support a lower, Asia-specific BMI cutoff for identifying a clinically meaningful lean phenotype [15]. A large nationwide Asian cohort study has also shown that, despite a normal BMI, lean MASLD carries a liver-related event risk comparable to non-lean MASLD (adjusted hazard ratio (aHR) 1.35, 95% CI 0.87-2.08) and significantly higher than that of a normal-liver control population (aHR 5.94, 95% CI 3.95-8.92), although its cardiovascular event risk is lower than that of non-lean MASLD (aHR 0.73, 95% CI 0.64-0.84) [18]; this underscores that the lean phenotype does not confer a benign prognosis and supports the relevance of identifying NAFLD in lean diabetic patients such as those in the present cohort.

A notable finding was that mean HbA1c did not differ significantly between participants with and without fatty liver (p=0.674), suggesting that NAFLD can occur across a broad spectrum of glycemic control in this lean cohort. The sonographic severity grade of individual participants could not be independently verified against original imaging records, and the association between NAFLD severity and HbA1c is therefore not reported (see Limitations).

Dyslipidemia has been proposed as a contributor to lean NAFLD pathogenesis in prior literature; however, in this cohort, elevated serum triglycerides (>149 mg/dL) were not significantly associated with NAFLD. This nonsignificant result does not exclude a role for triglycerides in lean NAFLD, given prior evidence linking elevated triglycerides and atherogenic dyslipidemia with hepatic fat accumulation [19,20], but it was not confirmed in this sample and should be interpreted cautiously given the modest sample size.

This study did not capture use of GLP-1 receptor agonists, SGLT2 inhibitors, insulin, or statins. GLP-1 receptor agonists in particular have also been shown to reduce alcohol consumption in individuals with type 2 diabetes or obesity [21], which is relevant because alcohol intake is a key exclusion criterion for NAFLD/MASLD diagnosis; unrecorded medication use could therefore have influenced both the metabolic phenotype and the reliability of alcohol-based exclusion in this cohort. Future studies in this population should systematically record medication use, diabetes duration, and waist circumference to allow adjusted analysis of these factors.

Demographic analysis showed a higher proportion of NAFLD among male patients (43.8%) than in female patients (32.7%), consistent with existing literature reporting higher rates of lean NAFLD in male patients [22,23]. Younger lean patients with diabetes (≤30 years) also showed a higher proportion of NAFLD (66.7% of a small subgroup), mirroring trends in South Asian populations where metabolic dysfunction can present at an earlier age [24]; however, this subgroup was small and should be interpreted cautiously. 

Limitations

This study has several limitations. It was conducted at a single tertiary care center with a modest sample size (n=100), which may limit generalizability; the cohort combined inpatients (n=86) and outpatients (n=14) from one hospital, and referral patterns at this center may not reflect the broader population of lean individuals with diabetes, though NAFLD prevalence did not differ significantly between the two groups (37.2% vs. 42.9%, p=0.69). NAFLD was diagnosed using transabdominal ultrasonography rather than liver biopsy or magnetic resonance spectroscopy, which are more sensitive for detecting and staging hepatic steatosis; in particular, ultrasound has limited sensitivity for detecting mild hepatic steatosis, which may have led to underestimation of NAFLD prevalence in this cohort. Although ultrasound examinations were performed prospectively by a single radiologist blinded to clinical history, diabetes treatment, and laboratory results, no second independent reader was used, so inter-observer variability in grading cannot be excluded; formal reliability assessment was not performed, and individual severity grades could not be independently re-verified against the original imaging records, so the association between NAFLD severity and HbA1c is not reported. Diabetes type was established on clinical grounds alone, without autoantibody or C-peptide testing (not performed for cost reasons), so misclassification of atypical diabetes (e.g., latent autoimmune diabetes of adulthood) cannot be fully excluded. Diabetes duration, waist circumference, and use of GLP-1 receptor agonists, SGLT2 inhibitors, insulin, and statins were not systematically recorded and could not be reported. This study also did not adjust for potential confounders such as age, sex, BMI within the lean range, duration of diabetes, treatment status, hypertension, and lipid parameters, and reported associations should be interpreted as unadjusted, bivariate comparisons rather than independent or causal relationships. The grade 3 NAFLD subgroup was also small (n=4), limiting the precision of severity-specific estimates. Additionally, the cross-sectional design precludes establishing causality between glycemic control and NAFLD progression.

## Conclusions

NAFLD was prevalent in this cohort of lean diabetic patients, affecting over one-third of those studied. NAFLD occurred across a broad range of glycemic control and, in this unadjusted analysis, was significantly associated with elevated fasting blood glucose but not with triglycerides or HbA1c. These findings highlight the importance of considering NAFLD in lean individuals with diabetes, even in the absence of obesity, and support further evaluation of the role of targeted screening in this population, ideally with adjustment for confounders in future studies.

## Appendices

Study data collection proforma

Name:  

Age:

Sex:

IP or OP No. :

Date:

*Chief complaints*:

*History of present illness*:

*Comorbid conditions*:    Diabetes mellitus:        Hypertension:Hypothyroidism:       Previous/present vascular events: 

*Social History*: Alcohol:     Smoking:      Diet:

*General physical examination*:

           Pallor:                                                                              Pulse rate:

           Icterus:                                                                            Blood pressure:

           Clubbing:                                                                        Respiratory rate:

           Cyanosis:                                                                        Saturation (pO2):

           Lymphadenopathy:

           Oedema:

Systemic examination

Per Abdominal examination:

Respiratory system :

Cardiovascular system :

Central nervous system :

Investigations

a) Fasting Blood Sugar (FBS)

b) Postprandial blood sugar (PPBS)

d) Random blood sugar (RBS)

e) HbA1c

f)  USG Abdomen and Pelvis

g)  Fasting Lipid profile (FLP)

Inference:
